# Acute cardiac injury events ≤30 days after laboratory-confirmed influenza virus infection among U.S. veterans, 2010–2012

**DOI:** 10.1186/s12872-015-0095-0

**Published:** 2015-09-30

**Authors:** Alison Ludwig, Cynthia Lucero-Obusan, Patricia Schirmer, Carla Winston, Mark Holodniy

**Affiliations:** Centers for Disease Control and Prevention, assigned to Veterans Affairs Office of Public Health Surveillance and Research, 3801 Miranda Avenue (132), Palo Alto, CA 94304 USA; Veterans Affairs Office of Public Health Surveillance and Research, 3801 Miranda Avenue (132), Palo Alto, CA 94304 USA; Division of Infectious Diseases and Geographic Medicine, Stanford University, Palo Alto, CA 94303 USA

**Keywords:** Influenza, Human, Myocardial ischemia, Cardiovascular disease, Veterans health

## Abstract

**Background:**

Cardiac injury is a known potential complication of influenza infection. Because U.S. veterans cared for at the U.S. Department of Veterans Affairs are older and have more cardiovascular disease (CVD) risk factors than the general U.S. population, veterans are at risk for cardiac complications of influenza infection. We investigated biomarkers of cardiac injury characteristics and associated cardiac events among veterans who received cardiac biomarker testing ≤30 days after laboratory-confirmed influenza virus infection.

**Methods:**

Laboratory-confirmed influenza cases among veterans cared for at U.S. Department of Veterans Affairs’ facilities for October 2010–December 2012 were identified using electronic medical records (EMRs). Influenza confirmation was based on respiratory specimen viral culture or antigen or nucleic acid detection. Acute cardiac injury (ACI) was defined as an elevated cardiac biomarker (troponin I or creatinine kinase isoenzyme MB) >99 % of the upper reference limit occurring ≤30 days after influenza specimen collection. EMRs were reviewed for demographics, CVD history and risk factors, and ACI-associated cardiac events.

**Results:**

Among 38,197 patients with influenza testing results, 4,469 (12 %) had a positive result; 600 of those patients had cardiac biomarker testing performed ≤30 days after influenza testing, and 143 (24 %) had one or more elevated cardiac biomarkers. Among these 143, median age was 73 years (range 44–98 years), and 98 (69 %) were non-Hispanic white. All patients had one or more CVD risk factors, and 98 (69 %) had a history of CVD. Eighty-six percent of ACI-associated events occurred within 3 days of influenza specimen collection date. Seventy patients (49 %) had documented or probable acute myocardial infarction, 8 (6 %) acute congestive heart failure, 6 (4 %) myocarditis, and 4 (3 %) atrial fibrillation. Eleven (8 %) had non-cardiac explanations for elevated cardiac biomarkers, and 44 (31 %) had no documented explanation. Sixty-eight (48 %) patients had received influenza vaccination during the related influenza season.

**Conclusion:**

Among veterans with laboratory-confirmed influenza infection and cardiac biomarker testing ≤30 days after influenza testing, approximately 25 % had evidence of ACI, the majority within 3 days. Approximately half were myocardial infarctions. Our findings emphasize the importance of considering ACI associated with influenza infection among patients at high risk, including this older population with prevalent CVD risk factors.

## Background

Every year in the United States, influenza results in an estimated 200,000 hospitalizations and 3,300–49,000 deaths [1, 2]. Although pneumonia is the most common complication of influenza infection, influenza is also associated with exacerbations of underlying medical conditions, including chronic cardiac diseases (e.g., congestive heart failure and atrial fibrillation) [3, 4]. In addition, influenza infection can be associated with experiencing cardiac injury, including myocardial infarction (MI), myocarditis, and congestive heart failure [5–11]. The increased risk for cardiovascular morbidity and mortality, particularly among people aged ≥85 years, has been described in observational studies regarding winter increases in excess deaths associated with influenza epidemics [12–16]. Ecologic studies of the association between the timing of influenza circulation and cardiac injury have also described an increase in incidence of acute MI during influenza epidemic weeks, compared with non-epidemic weeks [17, 18]. Case-control, retrospective cohort, and randomized controlled trial investigations have also illustrated an increased risk for certain types of cardiac injury, including acute MI or hospital admission for heart failure during influenza seasons; such studies have included documented receipt of influenza vaccine and a decreased risk for vascular events with use of antivirals for influenza infection among patients with history of cardiovascular disease [19–23].

In fiscal year 2012, approximately 8.8 million U.S. veterans received treatment from the U.S. Department of Veterans Affairs (VA) Veterans Health Administration (VHA) system [24]. VHA is the largest integrated healthcare system in the United States and provides comprehensive medical care to veterans through 151 medical centers and >1,500 associated community-based outpatient clinics, skilled nursing facilities, long-term care facilities, and other facilities. Because U.S. veterans receiving treatment from VHA are older and have more cardiovascular disease risk factors than the general U.S. population, veterans might be particularly vulnerable to cardiac complications of influenza infection. In 2010, the median age of male veterans was 64 years, compared to 36 years in the general U. S. population [25, 26]. Veterans also have a notable burden of cardiovascular disease and cardiovascular risk factors. Richlie et al. reported that 80 % of veterans seeking care at a VHA facility had two or more cardiovascular risk factors (defined in that study as male sex, history of coronary heart disease (CHD), family history of CHD, decreased high-density lipoprotein, smoking, history of cerebrovascular or peripheral vascular disease, hypertension, obesity, or diabetes) [27]. These characteristics of older age and frequent cardiovascular risk factors indicate veterans might be particularly vulnerable to unhealthy outcomes associated with influenza infection, including cardiac complications [28, 29].

The VA Office of Public Health conducts routine surveillance to detect and track influenza-like illness and influenza infection among veterans receiving VHA care [30]. Specifically, the Office of Public Health uses data from electronic medical records and the VA Electronic Surveillance System for the Early Notification of Community-Based Epidemics (ESSENCE), including *International Classification of Disease, Clinical Modification*, 9^th^*Revision* (ICD-9-CM) codes and laboratory data, to conduct and report weekly influenza surveillance [31]. The frequency of cardiac injury associated with influenza infection among veterans, however, is unknown. Using the robust platform of existing routine influenza surveillance within the VHA system’s electronic records, we describe the frequency and characteristics of cardiac injury among veterans who had cardiac biomarker testing ≤30 days after laboratory-confirmed influenza diagnosis during the 2010–2012 influenza seasons.

## Methods

We included 154 VHA facilities with laboratory capacity for influenza testing, including reference laboratory testing, and that also had cases of laboratory-confirmed influenza infection reported during October 3, 2010–December 31, 2012, the period for which data were available. Electronic influenza laboratory results for 139 facilities were available through ESSENCE. For 15 facilities where electronic influenza laboratory data were unavailable in ESSENCE, we reviewed hospitalizations and outpatient visits in ESSENCE with an ICD-9-CM diagnosis code of influenza (ICD-9-CM 487 or 488) and performed manual chart reviews of EMRs to determine if influenza testing had been performed. All influenza testing on respiratory samples was included; influenza serum antibody testing was excluded. Laboratory-confirmed influenza infection was defined as occurring in a patient in whom influenza virus was detected in a respiratory sample on the basis of direct fluorescence, enzyme immunoassay, nucleic acid detection, polymerase chain reaction assay, or viral cultures.

To identify cases of cardiac injury, we analyzed electronic laboratory records of patients with laboratory-confirmed influenza infection to identify veterans who had cardiac biomarker tests performed ≤30 days after a laboratory-confirmed influenza infection specimen collection. The 30-day period was chosen as a reasonable length of time to seek cardiac injury events that might be temporally linked to influenza infection [10]. The data source for cardiac biomarker results was the VA Corporate Data Warehouse (VA CDW), a national electronic data repository derived from the VA’s EMR system and administrative systems; the VA CDW is used for clinical and operational research, quality programs, and surveillance activities. A case of acute cardiac injury (ACI) was defined as an elevated cardiac biomarker (troponin I level or creatinine kinase isoenzyme, CK MB) value of >99 % of the upper reference limit and occurring ≤30 days after the date of laboratory-confirmed influenza specimen collection. This threshold for elevation was chosen as a standard reference for evidence of myocardial injury [32].

We categorized ACI-associated events by identification of documented physician diagnoses. Physician diagnoses were abstracted from EMR progress notes associated with the date of positive cardiac biomarker result, including where applicable, cardiology consult notes, and in the case of admissions, inpatient progress notes and discharge summaries associated with dates of admission during which the positive cardiac biomarker results occurred. Physician diagnoses of ACI-associated events were categorized as (1) ST segment elevation MI (STEMI); (2) non-ST segment elevation MI (NSTEMI) or probable NSTEMI (we defined NSTEMI as documentation of “NSTEMI” or “non-ST segment elevation MI” and probable NSTEMI as documentation of “supply demand mismatch” or “demand ischemia” without an explicit diagnosis of NSTEMI); (2) acute congestive heart failure; (3) myocarditis; (4) atrial fibrillation, including recurrence of previously diagnosed atrial fibrillation and excluding unchanged chronic atrial fibrillation; (5) other cardiac event, including hypertensive emergency; (6) non-cardiac diagnoses, including chronic renal failure; and (7) no documented physician explanation for the cardiac biomarker result.

From EMR progress and admission notes, we abstracted the following variables for all patients at the time of laboratory-confirmed influenza specimen collection: (1) demographics; (2) cardiovascular disease risk factors, defined as physician diagnoses of hypertension, diabetes, hyperlipidemia, pharmacologic treatment for hypertension, hyperlipidemia, or diabetes at time of influenza test, any history of tobacco use, or obesity (body mass index ≥30) (male sex was not included as a cardiovascular disease risk factor); (3) history of cardiovascular disease, defined as physician diagnosis of cerebral or peripheral vascular disease, coronary heart disease, MI, atrial fibrillation, or congestive heart failure; (4) acute comorbidities at time of influenza specimen collection, defined as acute non-cardiac presenting diagnoses aside from influenza documented by a physician (e.g., pneumonia, exacerbation of chronic obstructive lung disease, or acute renal failure); (5) severity of influenza-related illness as described by hospital admission, length of stay, and need for intensive care unit admission, including mechanical ventilation; (6) vital status at 30 days after influenza laboratory test, with date of death collected in applicable cases and (7) non-steroidal anti-inflammatory and aspirin use at time of influenza test. Date of influenza symptom onset (non-specific dates (e.g., notations of “a few days” or “several days” were excluded from the analysis), and the longest given day count was used when a range was given (e.g., 4 days would be recorded if “3–4 days” was documented). Cause of death was not routinely available and therefore was not abstracted. Electrocardiogram (EKG) reports associated with the date of positive cardiac biomarker result were reviewed to identify physician-reported changes compared with baseline. These were abstracted from final EKG reports, not from physician initial interpretations documented in progress notes, admission, or discharge notes. We also abstracted influenza vaccination history for the recommended influenza vaccination period corresponding to influenza diagnosis. We defined recommended influenza vaccination period as August 1 of the year of influenza diagnosis (the earliest date at which seasonal influenza vaccines are available within the VHA system) until 2 weeks before influenza test; we excluded vaccination if it had occurred <2 weeks before influenza diagnosis, assuming the patient’s immune response to the vaccination was incomplete. Documented receipt of vaccination at outside facilities in the EMR was included in vaccination history. Outside facility records were not available for review. Finally, we abstracted prescription details regarding initiation of antiviral treatment after positive influenza test. Data on antiviral therapy received at outside facilities was not available.

We calculated the time from influenza diagnosis to cardiac injury by using the interval between date of specimen collection of first positive influenza laboratory result and the date of first positive cardiac biomarker result. For patients with multiple elevated cardiac enzyme results or multiple positive influenza results, the first date with a positive cardiac enzyme result or positive influenza result was considered the reference date for time interval calculations. We calculated time from influenza test specimen collection to initiation of antiviral treatment for all patients who received antiviral therapy and time from influenza symptom onset to antiviral treatment for patients with a specified symptom onset date. Patient age was recorded on the basis of the date of influenza specimen collection. This study was reviewed for human subjects protection by the Centers for Disease Control and Prevention and determined to be non-research.

## Results

During October 2010–December 2012 (the period for which data was available), a total of 38,197 patients had influenza laboratory tests performed and captured in ESSENCE. Among those, 4,278 (11 %) had laboratory-confirmed influenza virus infection. Among the 15 facilities that do not have electronic laboratory reporting to ESSENCE, an additional 191 patients with laboratory-confirmed influenza virus infection were identified through manual chart review, for a total of 4,469 (12 %) patients with laboratory-confirmed influenza virus infection. Six hundred (14 %) veterans with laboratory-confirmed influenza virus infection also had a cardiac biomarker test performed ≤30 days after influenza specimen collection, of whom 143 (24 %) had a troponin or CK-MB result >99 % of the upper reference limit for their test (different facilities used different tests with difference reference ranges). In this descriptive study focusing on patients with both influenza and elevated cardiac biomarkers, we limited our analysis to these 143 patients and did not review data from the 457 patients with laboratory-confirmed influenza diagnosis and normal cardiac biomarker results.

### De*mographic characteristics*

Among 143 patients with laboratory-confirmed influenza virus infection and a cardiac biomarker result >99 % of the upper reference limit, the median age was 73 years (range 44–98 years); 98 (69 %) were non-Hispanic white; 32 (22 %) were non-Hispanic black and 2 (1 %) were female (Table 1). All patients had at least one risk factor for cardiovascular disease. One hundred three (72 %) had two or more risk factors and 28 (20 %) had three or more risk factors. Ninety-eight patients (69 %) had documented history of cardiovascular disease (Table 1).

**Table 1:** Clinical and demographic characteristics of U.S. veterans with acute cardiac injury ≤30 days after laboratory-confirmed influenza virus infection, October 2010–December 2012 (*n* = 143)

Characteristic	No.	%
Sex		
Male	141	99
Female	2	1
Age group (yrs)		
18–44	1	<1
45–64	38	27
65–84	71	50
≥85	33	23
Race/ethnicity		
Non-Hispanic white	98	69
Non-Hispanic black or African American	32	22
Hispanic or Latino	6	4
Not reported	7	5
Cardiovascular risk factors^a^		
Hyperlipidemia	118	83
Diabetes	67	47
Hypertension	128	90
Any history of tobacco use	94	66
Body mass index ≥30	49	34
History of cardiovascular disease^a^		
Cerebral or peripheral vascular disease	43	30
Coronary artery disease	57	40
Atrial fibrillation	30	21
Congestive heart failure	43	30

^a^The cardiovascular risk factor and history of cardiovascular disease categories are not mutually exclusive, and patients can have multiple characteristics; hence, the percentages do not add to 100

### Clinical characteristics

Among the 105 (73 %) patients with influenza symptom onset date recorded, the median time from onset to influenza specimen collection was 2 days (range 0–9 days), with 85 (81 %) patients having influenza specimens collected ≤3 days after symptom onset. Among the 105 patients with symptom onset date recorded, 59 (56 %) had positive cardiac biomarker testing ≤3 days after influenza symptom onset. The median time from influenza symptom onset to ACI was 3 days (range 0–25 days).

**Fig. 1:**
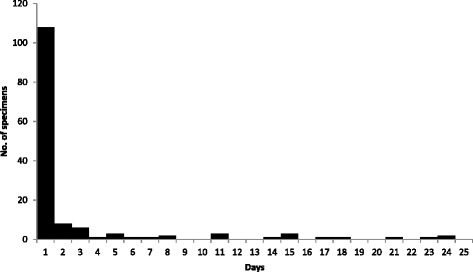
Number of days from influenza specimen collection to acute cardiac injury among U.S. veterans, October 2010–December 2012 (*n* = 143)

Among the total 143 patients with laboratory-confirmed influenza virus infection and ACI, the median time from influenza specimen collection to ACI was 1 day (range 0–24 days), and 123 (86 %) occurred ≤3 days after influenza specimen collection (Fig. 1). No STEMI events were documented. Thirty-six (25 %) patients had an NSTEMI documented in the medical chart ≤30 days after influenza specimen collection. In addition, 34 (24 %) were categorized as probable NSTEMI. Eighteen (13 %) patients had documented cardiac diagnoses (e.g., congestive heart failure or new-onset atrial fibrillation) (Table 2). Eleven (8 %) were categorized as non-cardiac diagnosis, of which four where attributed to chronic renal disease. Forty-four (31 %) were categorized as no documented physician explanation for the cardiac biomarker result. Of these 44 patients, 22 (50 %) had repeat cardiac biomarkers performed ≤24 h after initial positive cardiac biomarker result. Among those 22, a total of 16 had decreased to a ≤99 % threshold for test positivity and six remained elevated. Among the 127 (89 %) who had finalized EKG interpretation documented at time of positive cardiac biomarker result, 32 (25 %) had any EKG change, compared with prior EKG interpretations.

**Table 2:** Clinical characteristics of acute cardiac injury among 143 influenza-infected U.S. veterans, October 2010–December 2012

Characteristic	No.	(%)^a^
Cardiac injury type		
NSTEMI	36	25
Probable NSTEMI	34	24
Acute congestive heart failure exacerbation	8	6
Myocarditis	6	4
Atrial fibrillation	4	3
Non-cardiac explanation	11	8
No documented explanation	44	31
Total	143	100

NSTEMI, non-ST segment elevation myocardial infarction

^a^Percentage does not total 100 because of rounding

Among the total 143 patients with influenza virus infection and ACI, 89 (62 %) had physician documentation of having one or more additional acute non-cardiac presenting comorbidities. Among these 89 patients, the three most common acute non-cardiac presenting comorbid diagnoses were bacterial pneumonia (69 %), chronic obstruction pulmonary disease (COPD) exacerbation (40 %), and acute renal failure (35 %); 35 had two or more of these acute non-cardiac comorbid diagnoses. Among the 70 patients with probable or documented NSTEMI, 42 (60 %) were receiving pharmacologic treatment with aspirin and 45 (64 %) with a statin at the time of laboratory-confirmed influenza diagnosis. Data regarding pharmacologic treatment with aspirin or a statin at the time of NSTEMI diagnosis was not collected.

### Clinical outcomes

Among the total 143 patients with influenza virus infection and ACI, 134 (94 %) were admitted to the hospital ≤30 days after laboratory-confirmed influenza virus infection specimen collection. Among the 134 patients admitted, the median length of stay was 6 days (range 1–106); 44 (33 %) were admitted to an intensive care unit. Among those 44 patients, 25 (57 %) required mechanical ventilation. The median length of intensive care unit (ICU) stay was 6 days (range 2–30).

**Fig. 2:**
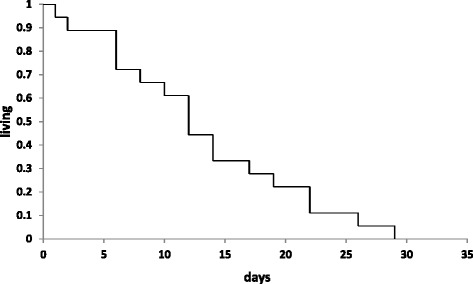
Time to death after positive cardiac biomarker among U.S. veterans, October 2010–December 2012 (*n* = 18)

Eighteen (13 %) of 143 patients died of any cause ≤30 days after laboratory-confirmed influenza virus infection specimen collection. Twelve of 18 (67 %) died ≤14 days after laboratory–confirmed influenza virus infection specimen collection (Fig. 2). Eleven (61 %) of those who died received a diagnosis of NSTEMI or probable NSTEMI ≤30 days after laboratory-confirmed influenza virus specimen collection.

### Vaccination and antiviral treatment

Sixty-eight of 143 (48 %) patients with influenza virus infection and ACI had documented receipt of influenza vaccination during the season of confirmed influenza virus infection and ≥2 weeks before the date of influenza specimen collection. Among 134 patients who were admitted, 71 (53 %) were unvaccinated. Of the 18 who died ≤30 days after laboratory-confirmed influenza infection, 9 (50 %) were unvaccinated. One hundred-eighteen of 143 (83 %) patients with influenza virus infection and ACI received influenza antiviral agents; all received oseltamivir. Among 105 patients with influenza symptom onset data available, median time from influenza symptom onset to initiation of influenza antiviral treatment was 2 days (range 0–18 days). Among 118 who had received influenza antiviral agents, 114 (97 %) received treatment ≤2 days after positive influenza specimen collection. Among 18 patients who died, 17 had received influenza antiviral therapy. Among ten deaths for which exact symptom onset date was available, five had received influenza antiviral treatment ≤2 days after symptom onset.

## Discussion

Overall, among veterans cared for at the US Veterans Health Administration diagnosed with influenza virus infection who also had cardiac biomarker testing performed ≤30 days after laboratory-confirmed influenza infection specimen collection, approximately 25 % had evidence of ACI. Among patients with influenza virus infection and ACI, >80 % of ACI was diagnosed ≤3 days after influenza specimen collection. Approximately half of ACI-associated events were probable or documented NSTEMI. Older veterans with risk factors for cardiovascular disease who were tested for both influenza and cardiac injury demonstrated a high prevalence of cardiac complications at or near the time of influenza diagnosis.

Our case-series study is unique in the inclusion of both laboratory-confirmed influenza cases and laboratory-confirmed cardiac injury findings. Prior studies investigating the association between influenza infection and cardiac injury have typically used diagnosis codes or clinical syndrome coding for both definitions [10, 20]. Previous studies have confirmed MI clinical diagnosis by using cardiac biomarkers, but none identified cardiac injury of all patients with positive biomarkers. Similarly, case-control studies have compared patients with cardiac injury, most often MI, with population-based control subjects to understand the relative effect of non-specific acute respiratory infection (ARI) on case status [20, 33–35]. These investigations used less-specific exposure definitions based on symptoms and physician diagnosis taken from administrative data (e.g., diagnosis codes), compared with our use of laboratory-confirmed influenza diagnosis, and report the odds ratios of MI associated with ARI ranging from 2.1 (95 % confidence interval (CI) 1.4–3.2) [34] to 3.6 (95 % CI 2.2–5.7) [20]. Because we identified a wider range of cardiac injury, compared with previous studies investigating the association between influenza infection and cardiac disease using diagnosis codes or clinical syndromes focused primarily on acute MI, our study provides a unique perspective on the role of influenza in cardiovascular disease. Although the diversity of cardiac manifestations associated with influenza infection have been described previously [3], our review contributes detailed diagnosis, morbidity, and outcome data among a population at risk for both complicated influenza infection and cardiovascular disease.

The fact that approximately one in seven veterans with laboratory-confirmed influenza had cardiac biomarker testing ≤30 days after their infection, and of those tested, approximately 25 % had evidence of ACI, suggests that there may be a link between acute influenza infection and risk for cardiac injury events.

Interestingly, no STEMI events were identified, whereas approximately half were either probable or documented NSTEMI. These NSTEMI events frequently represented MI type 2 presentations, reflecting myocardial injury with necrosis secondary to an imbalance between myocardial supply and demand among critically ill patients [37]. These patients are still at high risk for unhealthy outcomes because even low-level increases in cardiac biomarkers without EKG changes have documented higher risk for death or MI [36, 37]. Cases of subclinical cardiac injury also might have been missed because of normal cardiac biomarkers. One recent investigation reported that, among 39 patients admitted with severe influenza A (H1N1) who received echocardiography during October 2009–March 2011, 28 (72 %) had abnormal ventricular function on echocardiography; troponin levels were low (mean troponin 0.03 ng/ml (range 0.01–0.3)), with only one markedly elevated in the setting of an arrhythmia, indicating that cardiac dysfunction associated with influenza infection might not always include elevation in cardiac biomarkers [38]. Approximately half of the patients with probable or documented NSTEMI in our analysis were being treated with aspirin or statins at the time of laboratory-confirmed influenza diagnosis, which might have mitigated their risk for acute thrombotic events [39, 40].

We determined that ACI was frequently close to the time of influenza symptom onset and influenza testing. This short time interval between influenza onset and cardiac injury is consistent with the timing of cardiac injury reported in previous investigations that used less-specific markers for influenza infection (e.g., acute respiratory infection ICD-9-CM coding) and looked specifically at MI [34, 35]. Two self-controlled case-series and one case-control study described the highest risk for acute MI during the first 3–5 days immediately after respiratory infection [34, 35]. These investigations used the date of clinic diagnosis for the exposure variable definition, perhaps a less-specific date than date of symptom onset that we were able to identify from chart review for a subset of patients. Similar to previous investigations, our description of ACI at time of influenza symptom onset and diagnosis indicates that, among older patients with cardiovascular risk factors, clinicians should consider the acute period of influenza symptom onset and diagnosis as a potentially high-risk period for cardiac complications. Additionally, physicians might consider optimizing cardiac risk factor mitigation (e.g., pharmacologic and behavior management) for such patients.

Although the majority of patients with influenza virus infection and ACI, including in this analysis, received influenza antiviral agents, approximately half did not receive them within the recommended 2-day period after influenza symptom onset [41]. We did not have access to date of clinic presentation for this analysis, and possibly, these patients presented for care later than those who were treated ≤2 days after influenza symptom onset and may not have been offered treatment. The Advisory Committee on Immunization Practices (ACIP) recommends that influenza antiviral treatment be offered to any patient with confirmed or suspected influenza who is hospitalized, has severe or complicated illness, or is at higher risk for influenza complications, including adults aged ≥65 years and those with chronic underlying conditions, including cardiovascular disease [41]. ACIP also recommends routine influenza vaccination for all persons aged ≥6 months that do not have contraindications to vaccination [42]. Observational studies have reported that treatment ≤5 days after influenza symptom onset among severely ill populations has clinical benefits that include reduced mortality and duration of symptoms and complications from influenza. A meta-analysis conducted by Hsu et al., which reviewed >1,600 hospitalizations and 150,000 patients, supported early use of antivirals ≤48 h after symptom onset and confirmed mortality, hospitalization length, intensive care unit admission, and respiratory failure reduction with antiviral use ≤5 days among such severely ill hospitalized patients as those identified in our analysis [43]. No investigations specifically addressing the role of antivirals and ACI were identified in our literature review.

Although certain vaccinations might have been administered at outside facilities and not recorded in the VA medical record system, given the goal of 90 % influenza vaccination rate for veterans cared for by the VA, our finding of a <50 % vaccination rate among affected patients illustrates a noteworthy opportunity for improvement in preventative services [44].

A potential limitation of our study is that certain cardiac events might have been missed because of differences in cardiac biomarker testing protocols or atypical clinical presentations where testing was not performed, as well as the possibility that veterans sought care at non-VA hospitals with no corresponding cardiac biomarker results captured in VA data. In addition, cardiac-biomarkers typically reflect ischemic injury and as such, non-ischemic ACI events such as acute congestive heart failure and atrial fibrillation may have been underestimated. By starting with positive cardiac enzymes to identify patients for review, we captured cardiac events that would have been missed by looking at ICD-9 codes alone, as the majority of previously published studies have done. In particular, probable NSTEMI cases, which represent substantial cardiac events, would have been missed if examining diagnosis codes alone because these cases did not have a specific acute cardiac injury type documented in the medical record. Because veterans can receive care at outside facilities and not all this information is recorded in the VA’s EMR system, we also were likely missing vaccination events, influenza infection, and ACI events. As we only examined veterans who were tested for influenza, we likely selected for a sicker group who might have other characteristics different from the general population or other veterans. Similarly, because of time and resource limitations in this descriptive study designed for timely feedback for public health use by VHA, we did not review the 457 cases with laboratory-confirmed influenza infection and normal cardiac biomarker testing for comparison. Nevertheless, we believe that our analysis, based on laboratory diagnosis of both influenza and ACI, provides a unique description of ACI-associated events after influenza infection.

## Conclusion

Acute cardiac injury, most commonly occurring during the first week of influenza virus infection and diagnosis, was identified in approximately 25 % of patients with laboratory-confirmed influenza who received cardiac biomarker testing. These events were associated with a high prevalence of cardiac history and risk factors, hospitalization, and a >10 % all-cause mortality rate at 30 days. Influenza antiviral treatment and vaccination were not uniformly received by this population at high risk and are notable missed public health opportunities. Given the high prevalence of cardiac comorbidities and influenza risk among older U.S. veterans, this analysis highlights the importance of (1) considering concurrent cardiac events when diagnosing influenza; (2) encouraging yearly influenza vaccination among older U.S. veterans; and (3) aggressively identifying and modifying possible cardiovascular risk factors when diagnosing influenza, ensuring prompt treatment with influenza antiviral medication.

## Abbreviations

ACI: Acute cardiac injury
ACIP: Advisory Committee on Immunization Practices
ARI: Acute respiratory infection
CHD: Coronary heart disease
CVD: Cardiovascular disease
EKG: Electrocardiogram
EMR: Electronic medical record
ESSENCE: Electronic Surveillance System for the Early Notification of Community-Based Epidemics
ICD-9-CM: International Classification of Disease, Clinical Modification, 9^th^ Revision
MI: myocardial infarction
NSTEMI: non-ST segment elevation MI
STEMI: ST segment elevation MI
VA: Veterans Affairs
VA CDW: VA Corporate Data Warehouse
VHA: Veterans Health Administration
